# Excess Provisional Extracellular Matrix: A Common Factor in Bicuspid Aortic Valve Formation

**DOI:** 10.3390/jcdd8080092

**Published:** 2021-08-04

**Authors:** Christine B. Kern

**Affiliations:** Department of Regenerative Medicine and Cell Biology, 171 Ashley Avenue, Medical University of South Carolina, Charleston, SC 29425, USA; kernc@musc.edu

**Keywords:** bicuspid aortic valve, aortic valve, versican, aorta, outflow tract, neural crest, NOTCH1

## Abstract

A bicuspid aortic valve (BAV) is the most common cardiac malformation, found in 0.5% to 2% of the population. BAVs are present in approximately 50% of patients with severe aortic stenosis and are an independent risk factor for aortic aneurysms. Currently, there are no therapeutics to treat BAV, and the human mutations identified to date represent a relatively small number of BAV patients. However, the discovery of BAV in an increasing number of genetically modified mice is advancing our understanding of molecular pathways that contribute to BAV formation. In this study, we utilized the comparison of BAV phenotypic characteristics between murine models as a tool to advance our understanding of BAV formation. The collation of murine BAV data indicated that excess versican within the provisional extracellular matrix (P-ECM) is a common factor in BAV development. While the percentage of BAVs is low in many of the murine BAV models, the remaining mutant mice exhibit larger and more amorphous tricuspid AoVs, also with excess P-ECM compared to littermates. The identification of common molecular characteristics among murine BAV models may lead to BAV therapeutic targets and biomarkers of disease progression for this highly prevalent and heterogeneous cardiovascular malformation.

## 1. Introduction

A bicuspid aortic valve (BAV), as opposed to the normal tricuspid arrangement, is the most common cardiac malformation and is found in approximately 0.5% to 2% of the population [1,2]. BAVs are present in 50% of isolated severe aortic stenosis cases that require surgery and can lead to bacterial endocarditis [3]. BAVs are also an independent risk factor for ascending aortic aneurysms, which can lead to rupture (dissection) and sudden death. This association may be a result of their common developmental origin and/or abnormal changes in hemodynamic forces due to a BAV [4]. Currently, there are no therapeutics to treat BAVs and the heterogeneity of the disease makes it challenging to design therapeutic approaches and to predict the need for surgical intervention [5]. The relatively few genes linked to human BAV leave the underlying genetics involved in BAV formation poorly defined. However, the use of genetically modified murine models with BAVs has given insight into the cell and molecular interactions required for normal aortic valve (AoV) development. Characterization and collation of murine models with BAV provides the opportunity to discover common molecular signatures among seemingly diverse murine BAV models. Since BAVs appear to have a complex origin and exhibit an unpredictable disease progression, the discovery of common characteristics among murine BAV models may reveal novel therapeutic targets that are effective for a significant cohort of BAV patients.

## 2. Development of the Aortic Valve

The AoV develops from the cardiac outflow tract (OFT), an embryonic structure that also gives rise to the pulmonary valve (PV), the ascending aorta, and the pulmonary artery [6]. OFT remodeling refers to the complex series of growth and morphological transformations required to form the mature cardiac structures that are derived from the initial OFT. In fact, the majority of cardiovascular defects, such as changes in valve cusp number, overriding aorta, ventricular septal defects, double outlet right ventricle, hypoplastic left heart syndrome, and aortic coarctation arise from aberrant OFT remodeling [7,8,9,10].

**Figure 1:**
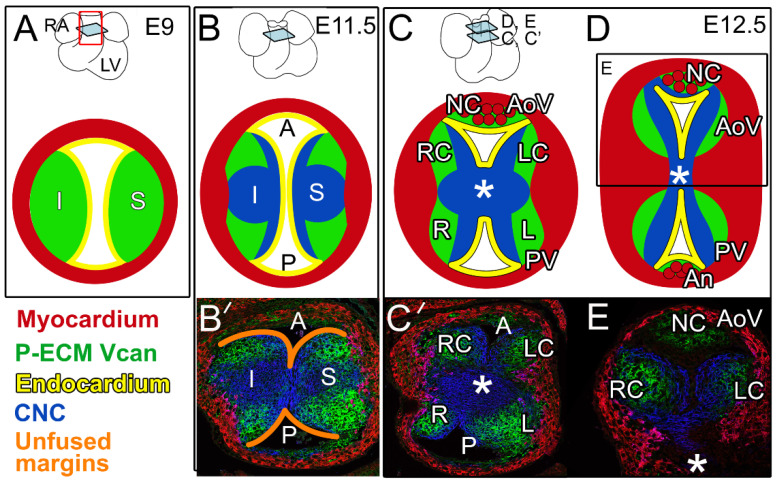
Provisional extracellular matrix (P-ECM, green) is downregulated during OFT remodeling and exhibits a complementary pattern to cardiac neural crest (blue). Sections of remodeling OFT shown for embryonic day 9 (E9) in panel (**A**), E11.5 in panels (**B**,**B’**), and E12.5 in panels (**C**,**C’**,**D**,**E**). Whole hearts are shown on the top row in panels (**A**–**D**). Light blue squares on the whole heart schematics indicate the relative position of the OFT cross-sections in each panel: RA—right atrium, LV—left ventricle; red rectangular outline in A indicates OFT region of the heart. Schematics of cross-sections depicting the remodeling OFT (**A**–**D**): Red—myocardium; yellow—endocardium; green—versican (Vcan) indicative of P-ECM; S—superior cushion; I—inferior cushion; orange outlines (**B’**)—unfused margins of the S and I fused cushions; A—aortic channel; P—pulmonic channel; L, LC—left and left coronary cusps of the pulmonary valve (PV) and aortic valve (AoV), respectively; R, RC—right and right coronary cusps of the PV and AoV, respectively; NC—non-coronary cusp of the AoV; An—anterior cusp of the PV; *—aorticopulmonary septum; red circles (**C**,**D**) denote myocardial lineage of Tnnt2-Cre origin of the NC and An cusps. Immunolocalization of OFT cross-sections (**B’**,**C’**,**E**); blue-Wnt1-Cre; EGFP indicates CNC lineage, green—intact Vcan, red—myocardium (smooth muscle actin).

Initially, at embryonic day 9 (E9), the cardiac OFT is a simple tube composed of an outer myocardial sleeve formed by the migration of cells from the secondary heart field (SHF) [11,12,13,14], and lined by specialized endothelial cells referred to as the endocardium (Figure 1A). In the OFT, blood flow in the early embryo is controlled by opposing endocardial cushions denoted as inferior (I) and superior (S) (Figure 1A,B). Endocardial cushions are formed by the secretion of ECM from adjacent myocardial and endocardial cells, and are often referred to as cardiac jelly. Cardiac jelly is provisional ECM (P-ECM) comprising the large aggregating proteoglycan, versican (Vcan), that is required for endocardial cushion formation [15,16] (Figure 1, green). Vcan binds to other ECM components, including hyaluronan, to generate the P-ECM architecture [17] that provides the viscoelastic properties of the endocardial cushions and serves as a substratum for mesenchymal cells from multiple lineages. Mesenchymal cells of neural crest origin (cardiac neural crest, CNC) migrate from the hindbrain into the distal OFT and populate the endocardial cushions beginning at approximately E9.5. As OFT remodeling progresses, CNC cells proliferate, condense, and migrate proximally toward the right ventricle [18,19,20]. OFT cushions in the proximal region are also composed of mesenchymal cells that arise from an endocardial to mesenchymal transformation (EndoMT). By approximately E11.5, the major OFT cushions join and fuse at the midline; this fusion separates the common OFT into the separate pulmonary and aortic channels (P and A in Figure 1A,B,B’,C’).

After fusion of the major OFT cushions (Figure 1B,C), their rounded edges remain separate (Figure 1B’, orange outlines), and are the precursors of the right coronary (RC) and left coronary cusps (LC) of the AoV on the posterior side and the left (L) and right (R) cusps of the PV on the anterior side (Figure 1C,C’). The CNC and EndoMT lineages [21,22,23,24] exhibit complementary patterning that is highly reproducible in the OFT cushions and developing cusps [21]. Recent work revealed that the CNC lineage was also complementary to Vcan localization in the P-ECM (Figure 1, blue vs. green) [21]. While there is still debate over the precise contribution of the SHF [12,13,14], lineage tracing experiments and conditional deletion strategies have revealed cross-talk among the different populations of prevalvular mesenchymal cells [23].

In coordination with the fusion of the major OFT cushions, the intercalated cushions develop at right angles to the midline fusion, and give rise to the non-coronary cusp (NC) of the AoV and anterior (An) cusp of the PV (Figure 1C,D) [21]. In the developing AoV, formation of the intercalated cushion involves invagination of the endocardial lining into the RC prevalvular cushion; however, the majority of the cells that contribute to the intercalated cushions are derived from the cTnnt2-Cre myocardial lineage [21,25,26]. The intercalated cushion of the PV forms from an inward expansion of cTnnt2-Cre lineage cells and develops independently of the R and L prevalvular cushions. In mice, the cTnnt2-Cre myocardial lineage predominates over the CNC- and EndoMT-derived cells in the intercalated cushion-derived cusps of the AoV and PV. It remains unclear how cell lineage patterning affects normal and abnormal cusp morphologies including the formation of BAVs. (Henderson et al. describe the potential origins of malformed AoVs in a complementary and thorough developmental review [25]).

As the AoV and PV are formed and sculpted throughout OFT remodeling, a subset of myocardial cells [26], as well as a portion of CNC cells, differentiate into smooth muscle cells (SMCs) and contribute to the aortic root and ascending aortic wall, respectively [20,27]. However, in spite of the developmental interdependence of the aortic valve and ascending aorta, few genes have been discovered that, when disrupted, give rise to both BAV and ascending aortopathies [28].

Although many critical developmental events have been defined in AoV formation, the stage, cell type, lineage, and molecular signaling events that generate BAVs largely remain a mystery. Moreover, the combination of BAV-linked human mutations that have been discovered to date, including *NOTCH1* [29], *SMAD6* [30], *GATA4* [31], *GATA5* [32], *GATA6* [33], *ROBO4* [34], *MAT2A* [35], and *ADAMTS19* [36], represent a relatively small number of BAV patients, leaving the genetic origins for the majority of individuals with BAV, unknown. The high frequency of BAV in the human population and relatively few genes linked to BAV indicate a complex etiology.

## 3. Murine Models of Human BAV-Associated Genes

To understand the underpinnings of BAV formation, approximately 30 murine models have been utilized with a BAV penetrance ranging from 8% to 91%, albeit with the majority less than 30% penetrant [37] (Table 1). The primary approach has been to generate mutations in mice that correspond to gene mutations found in BAV patient DNA. *NOTCH1* was the first gene linked to BAV in humans and since its linkage was elucidated over 15 years ago, there have been approximately 10 different mouse lines developed with mutations in the NOTCH1 pathway that give rise to BAVs [29,37,38,39,40,41,42,43].

### 3.1. Insight of BAV Formation from Mice with Mutations in Notch1 Pathway Genes

Investigations utilizing mice containing mutations that impact NOTCH1 signaling have given insight into AoV development. Embryonic death (E9.5) in mice with a *Notch1* homozygous deletion emphasizes the importance of NOTCH1 signaling in early development. Heterozygous *Notch1^+/−^* mice are viable but exhibit a low penetrant BAV (8%) [38]. The NOTCH1 transmembrane receptor is activated by ligands encoded by Delta-like 4 (*Dll4*), Jagged1 (*Jag1*), and Jagged2 (*Jag2*) [43]. The disruption of *Dll4* revealed NOTCH1 signaling is critical for EndoMT transition in OFT cushion formation [43]; this early role for NOTCH1-DLL4 is also consistent with the early embryonic deaths of *Notch1^−/−^* homozygotes [29]. NOTCH1-DLL4 activation is also regulated by endocytosis initiated from Mindbomb E3 Ubiquitin Protein Ligase 1 (*Mib1*); the conditional deletion of *Mib1* (*Nkx2.5^Cre^*; *Mib1^f/f^*) results in a low penetrant BAV (17%) [43]. In the post-EndoMT endocardium, when *Notch1* is conditionally inactivated (*Nfatc1^enCre^*;*Notch1^f/f^*), these mice exhibit BAV with 36% penetrance [43]. In addition, mice lacking endocardial JAG1, (Nfatc1*^enCre^*;*Jag1^f/f^*) display 25% penetrant BAV [43], while endocardial deletion of RBPJ, (*Nfatc1^enCre^*;*Rbpj^f/f^*) a transcription regulator of *Notch1*, results in the formation of a BAV with 33% penetrance [43]. Mice with the conditional deletion of genes encoding JAG1 and JAG2 using the myocardial cTnnt2-Cre (*Tnnt2^Cre^*;*Jag1^f/f^,Jag2^f/f^*) also develop BAV with a penetrance of 36% [41]. This work [41] and others [21,44] revealed that the NC of the AoV and An cusp of the PV, which are derived from the intercalated cushions, are primarily generated by myocardial cTnnt2-Cre lineage cells. Collectively, data using BAV models indicate NOTCH1 signaling is involved at many stages of AoV development and that the disruption of Notch1 signaling in the myocardial and endocardial lineages contributes to BAV formation.

### 3.2. BAV Formation Occurs in Mice with Mutations in Genes That Intersect with Notch Signaling

Additional BAV models were created with genetic perturbations in factors that intersect with NOTCH1 signaling, including nitric oxide synthesis 3, (NOS3), which activates NOTCH1 by promoting cleavage of the Notch IntraCellular Domain (NICD) and initiating the expression of NOTCH target genes. The compound mutant mice *Nos3^−/−^;Notch1^+/−^* exhibit a highly penetrant BAV (91%) [38] compared to the single mutants *Nos3^−/−^;Notch1^+/+^* (27%; [38]; 42% [38,45]) and *Nos3^+/+^;Notch1^+/−^* (8%) [38]. The deletion of *Gata5*, (*Gata5^−/−^)* a zinc finger transcription factor, which activates the expression of *Jag2* and *Nos3* and promotes cleavage of the NICD, results in BAV at 25% penetrance [46]. Conditional inactivation of *Gata5* in endothelial cells and their mesenchymal derivatives using the Tie2-Cre (*Tie2^Cre^*;*Gata5^f/f^*), gives a BAV penetrance of 21%, indicating the primary requirement of GATA5 is the endocardium [46]. *GATA5* variants are also associated with BAV in the human population [32,47]. The deletion of *Krox20^−/−^*, which encodes a zinc finger transcription factor that activates *Nos3*, gives rise to a murine model with a 27% penetrant BAV [48]. Since the discovery of the *NOTCH1* linkage in human BAVs [29], mice containing gene modifications that intersect with Notch signaling have expanded the ‘Notch1 AoV interactome’ in AoV development. (For comprehensive reports of Notch signaling in AoV development, consider reviews [5,49,50].

## 4. Collation of Murine BAV Data Identifies Common Pathological Intersections in BAV Formation

The comparison of BAV phenotypic characteristics between murine models is another tool that can advance our understanding of BAV pathogenesis (Table 1). We examined the current findings from all the murine models to date to identify the common molecular criteria that may intersect in BAV formation. The collation of murine BAV data may reveal molecular classification of BAVs that facilitates the identification of novel biomarkers and effective therapeutic targets for BAV pathogenesis.

### 4.1. Excess P-ECM Is a Common Pathological Finding in Murine BAV Development

**Figure 2:**
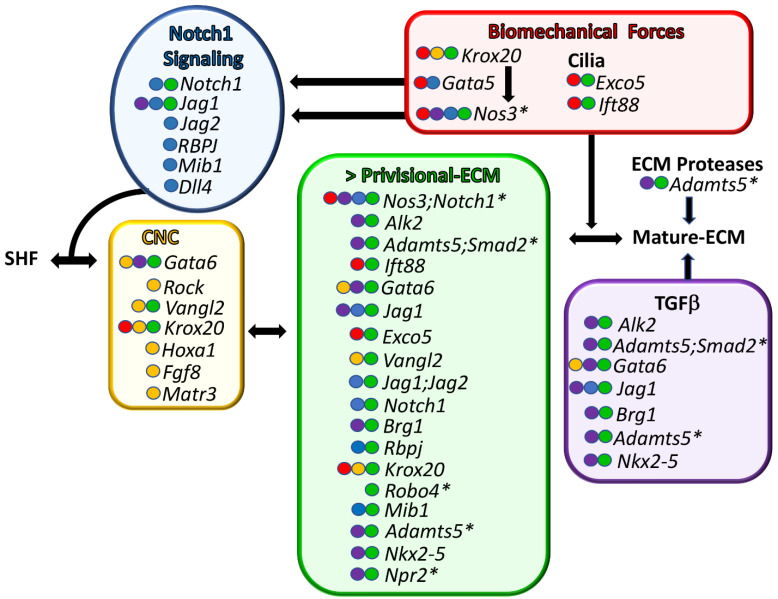
Common molecular characteristics of murine BAV models. Genes associated with BAV formation are shown. Each shared characteristic of BAV models is color-coded. Green—murine BAV gene mutations that result in excess provisional extracellular matrix (>P-ECM). Yellow—cardiac neural crest anomalies. Blue—NOTCH1 family BAV mutations. Red—genes associated with biomechanical signaling. Purple—TGFβ superfamily associated BAV genes. The colored circles in front of the gene name indicate shared BAV characteristics. Genes are listed from top to bottom, with the highest percent penetrant BAV at the top and the lowest at the bottom. In the cases where there are several different BAV models for the same gene, the gene name is listed in the order of the highest percentage of BAV observed between models. * denotes murine BAV models that exhibit ascending aortopathies in combination with BAV. SHF—secondary heart field lineage. Arrows represent gene product interactions as well as other interdependent connections.

Here, we show that collation of murine BAV data identified excess P-ECM (which includes thickened AoV cusps, increased Alcian blue staining, increased Vcan localization, and enlarged hinge regions) as a common factor in BAV formation (Figure 2, green) [23,34,38,41,43,48,52,54,55,56,57,61,62,63]. It was also noted that while the BAV frequency is low in many murine models (Table 1), the tricuspid AoVs from the mutant BAV mice exhibit larger and more amorphous cusps than their wild type littermates in the following models: *Isl1^Cre^*;*Vangl^f/f^* [41] *Nkx2.5^Cre^*;*Jag^f/f^* [43], *Nfatc1^enCre^*;*Jag1^f/f^* [43], *Nfatc1^Cre^*;*Ift88^f’/f^* [55], *Nfatc1^Cre^*;Fulmer *Exco5^f’/f^* [57], *Robo4^−/−^* [34], *Krox20^−/−^* [48], and *Nfatc1^Cre^*;*Brg1^f’/f^* [23]. There are various degrees of P-ECM characterization in publications of BAV models, these include Alcian blue staining, which identifies glycosaminoglycans that are present in hyaluronan and ECM proteoglycans, including Vcan (Table 1), the immunolocalization of Vcan, and the localization of hyaluronan using labeled hyaluronan binding protein (HABP) [15,16,59]. During normal AoV development, P-ECM components are replaced by mature ECM (M-ECM), such as fibrillar collagens, elastin, tenascin [23], and small leucine-rich proteoglycans (SLRP) [64,65]. The normal downregulation of P-ECM during AoV development is shown in Figure 1, with the reduction of Vcan (green) at each stage of OFT remodeling. Excess proteoglycans and hyaluronan are also associated with diseased adult AoVs in mice and humans [66,67,68,69], as well as in ascending aortopathies [53,70], a comorbidity of approximately half of BAVs in human patients [3]. In fact, tissues from end-staged diseased AoVs and ruptured aortic arteries contain a massive accumulation of ECM-aggregating proteoglycans [66,68,69,70,71]. Therefore, excess proteoglycans associated with P-ECM may contribute to both the origin of BAV malformation and adult disease progression.

### 4.2. Disruption of CNC Patterning Was Frequently Observed during BAV Formation in Murine Models

Altered CNC patterning (Figure 2) was also observed during BAV formation and included the following BAV models: *Isl1^Cre^;Gata6* [56], *Nos3^−/−^* [38], *Isl1^Cre^;Vangl2^f/f^*^,^ [41], *Rock^DN,^* [22], *Krox20(Egr2)^LacZ/LacZ,^* [48], *Hoxa1^GFP/GFP^* [58], *Hoxa3-I^Cre^;Fgf8^AP/N^*^,^ [59], and *Matr3^Gt-Ex13/Gt-Ex13^* [60]. The factors that govern neural crest guidance during AoV formation are not well understood, but remodeling of the P-ECM likely plays a significant role. Lineage tracing of CNC using the Wnt1-Cre transgene revealed a lack of CNC cells in Vcan-rich P-ECM (Figure 1, blue vs. green) [21]. Notably, CNC cells co-localize with an N-terminal cleavage fragment of Vcan generated by the ECM protease ADAMTS5, which is critical for normal semilunar valve formation [21]. Deficiency of CNC cells in the outflow endocardial cushions in *Pax3^cre/cre^* mice coincides with an increase in ECM during the late stages of valve development [72]. In addition, *Gata6*-deficient mice, with BAV penetrance ranging from 27 to 56%, have altered expression of Vcan-cleaving ECM proteases *Mmp2* and *Mmp9*. These mice also have thickened valves with increased Alcian blue staining [56]. An intersection of CNC and ECM remodeling among BAV models is emerging. The concept of lineage-dependent ECM composition in semilunar valve development was proposed by Akerberg et al. [23]. Nfatc1-based lineage tracing with *Nfatc1^Cre^*;*Brg1^f/f^* mice indicate that the mature ECM stratification of semilunar valves is conferred by the regulated patterning of the different lineages of mesenchymal cells; each lineage displays competency to produce specific ECM proteins [23]. In addition to NOTCH signaling, TGFβ [23,43,52,54,56,61,63], and biomechanically induced [38,46,48,55,57] gene clusters also emerged as common BAV characteristics (Table 1, Figure 2). The discovery of shared molecular criteria of BAV models may identify effective therapeutic targets for BAV patients that are grouped based on common pathological molecular signatures.

## 5. Proposed Set of Criteria to Evaluate and Compare Murine BAV Models

The collective examination of murine BAV models has reached a juncture that would be facilitated by the use of a standard rigorous set of characterization criteria that would maximize each model’s contribution to advancing BAV biology. Moreover, the technical advance of using endonuclease-mediated gene modifications will dramatically augment the number of additional animal models applicable to human BAV disease in the near future. The use of proposed BAV criteria for murine BAV models includes the following.

### 5.1. Evidence of a BAV Phenotype

The images required to demonstrate a murine BAV phenotype include three-dimensional (3D) views of the valve morphology. At early time points, this may involve a series of chemically stained histological sections shown alongside a 3D reconstruction derived from each section through the developing AoV [54]. Episcopic imaging [73] and scanning electron microscopy [74] are high-quality methods that will also reveal BAV morphology. At post-natal time points, microdissection and simple light microscopy can be sufficient [54]. Wild-type evaluation for BAV is imperative since there are different frequencies of BAVs among strains with significant genetic modifiers that promote BAV in the C57Bl/6 genotype [58,60,74].

### 5.2. Type of BAV Malformation (LC-RC, LC-NC, RC-NC), Presence or Absence of Raphes (That Indicate Partial Cusp Delineation)

There are reports that the type of BAV cusp ‘fusion’ may be indicative of its genetic origins during development [74,75,76]. The term ‘fusion’ is common in describing BAV, however, investigations reveal the bicuspid morphology is primarily due to the failure to fully form each cusp rather than a ‘fusion’ event between two distinct cusps. Comparison of the type of BAV malformations in mice reveals that a predominance of BAVs involve the NC cusp [52] (Table 1), which is different from human BAV patients, where the largest category involves RC-LC. There is also evidence that BAV morphology may not reflect the underlying genetics since mutations in *Gata5* cause different BAV types in humans vs. mice [32,56]. Moreover, BAV mouse models on identical genetic backgrounds, even within the same litter, can exhibit different BAV types [54] (Table 1). Therefore, caution should be taken when extrapolating morphological characterizations of BAV phenotypes from mouse to human [37,46,47] or that the type of cusp ‘fusion’ is indicative of shared etiologies. Nevertheless, the morphology of BAV is important to document and to determine differences and/or limitations of mouse models in understanding BAV development in human patients.

### 5.3. The Timepoint(s) of AoV Developmental Anomalies

One of the key aspects in determining the mechanisms involved in BAV formation is defining the stage(s) and morphological process(es) that are disrupted in murine BAV models. Comprehensive comparisons of staging between AoV development in humans and mice are described by Krishnan et al. [77]. Characterization of a BAV should include multiple timepoints in developing mice that are based on AoV developmental milestones—E11.5, E12.5, E14.5, and E17.5—to trace the origin of the developmental defect(s) and compare with littermate controls. By E11.5, the major OFT cushions have formed, and CNC cells have migrated to the proximal OFT region. In addition, OFT cushions begin to fuse at the midline, leaving the unfused margins as prevalvular primordia of the LC and RC. By E12.5, the intercalated cushions have developed and their relative positioning within the OFT (proximal for the AoV and distal for the PV), is apparent [21]. At E14.5, there is evidence of excavation, that is, thinning, and sculpting of the rather amorphous prevalvular cushions. At this stage, there is an indentation near the insertion of the cusp, referred to as the hinge region. During late gestation (E17.5–E18.5), the AoVs have a distinctive cusp-like morphology due to additional excavation. By E17.5, the valve cusp ECM is also organized into distinctive layers; the Vcan-rich ECM is now sequestered between fibrous elastic M-ECM and also localized in the more bulbous tip, as well as where the cusps are anchored. Characterization of each time point will enable comparisons between murine models and the extent to which malformations at each stage may correlate with BAV formation.

### 5.4. Cell Lineage Patterning Alterations during OFT Remodeling

Since the normal tricuspid AoV exhibits reproducible patterning of multiple lineages, tracing the different cell lineages and comparison among BAV murine models and their littermate controls will facilitate the understanding of lineage-specific roles in AoV cusp formation and their contribution to BAV. The Tdtomato-EGFP reporter, Gt(ROSA)26Sor^tm4(ACTB-tdTomato,-EGFP)Luo^/J, is a sensitive read-out of Cre recombination for cell lineage tracing. Popular and well-characterized Cre lineage drivers for the characterization of cell lineage contributions include cTnnt2 (myocardial, Tg(Tnnt2-cre)5Blh/JiaoJ), Wnt1 (CNC, 129S4.Cg-*E2f1^Tg(Wnt1-cre)2Sor^*/J), Tie2 (endothelial and EndoMT, Tg(Tek-cre)12Flv/J), and NfatC1^Cre^ (post EndoMT endocardial) [78].

### 5.5. Documentation of Abnormalities in the Ascending Aortic Artery (Aortopathies)

Since BAVs are an independent risk factor for aortopathies, examining the morphology of the developing aortic wall in the late stages of fetal development through aging adulthood will help in gaining insight into the comorbidities of BAV and aortopathies. Examples of characterizations of murine aortopathies are included in the work by Koenig et al. [51] and Dupuis et al. [53]. Of note, many current BAV models die prior to or shortly after birth, precluding the ability to investigate any potential role of the mutated gene in the development of the aortic wall.

### 5.6. Characterization of ECM Components Relevant to the Conversion of P-ECM to M-ECM

The conversion of the P-ECM to M-ECM in AoV development involves the reduction of the Vcan–HA matrix and the generation of a complex stratified ECM that includes a collagen-rich fibrous matrix, proteoglycan-rich, spongiosa and elastic fibrosa that integrates with aortic root tissues for strength and support. AoVs that exhibit an enlarged cusp morphology with reduced cell density are also indicative of excess P-ECM [63]. The P-ECM component HA is localized and quantified using HABP [79]. Vcan turnover, a key process in cardiac valve development is monitored by using a neo-epitope antibody that recognizes the cleaved fragment referred to as DPEAAE [63]. Intact Vcan can also be immunolocalized and quantified. Excess Vcan is indicative of altered P-ECM [54] and is reduced and restricted to the spongiosa in later stages of AoV development and in adult AoVs. Fibulin-1, which binds to Vcan and facilitates cleavage of proteoglycans by binding to ADAMTS proteases, is also highly expressed in the OFT cushion P-ECM [80,81,82]. Key M-ECM components include tenascin [23,83], periostin [23,54], fibrous collagens [54,84], and small leucine-rich proteoglycans [64,65]. Documenting changes in ECM components during AoV development in murine models of BAV will elucidate the interconnection of ECM remodeling and molecular signaling pathways involved in BAV formation. Overall, the spatiotemporal localization of ECM components of both the P-ECM and M-ECM is ongoing and will further define the ECM architecture that is required for normal AoV development and to prevent BAV malformations.

## 6. Discussion

The collation of murine BAV data described herein indicates that defective OFT remodeling due to dysregulated P-ECM to M-ECM conversion may be a common factor in BAV formation. Reduced turnover of Vcan, a critical component of remodeling P-ECM during AoV development, leads to malformations, including BAV [54]. These data suggest that a hallmark of diseased adult human BAV tissues, that is, massive accumulation of aggregating proteoglycans, may also be a common juncture in the origin of the BAV morphology at least in mice but also very likely in human BAV as well [66,68,69,70,71].

The collation of murine BAV data also indicates that a low penetrant BAV (less than 30% total mutants) in many murine models, is accompanied by abnormally thickened tricuspid AoV in the remaining mutated mice. The fact that the abnormal tricuspid AoV exhibited similar morphological and molecular characteristics as the BAV on identical genetic backgrounds during development indicates that given a slightly different biological context, the abnormal AoV may result in a BAV. Cardiac valves form in what may be the most biomechanically demanding developmental environment. Therefore, what we may consider as slight variances in utero of biomechanical forces may be a contributing factor in the developmental onset of the bicuspid vs. abnormal tricuspid morphology among genetically identical mutated mice. It is unclear how this partial BAV penetrance in murine BAV models translates to BAV patients, that is, how often do family members with the same BAV-associated gene mutation develop BAV, while others have tricuspid valves? These data indicate that family members containing a BAV-associated mutation may not always develop BAV, but on closer inspection, may have abnormal AoV or early-onset AoV calcification.

With such a heterogeneous disease, one of the major initiatives for determining the common factor(s) of BAVs in mice is the need for novel therapeutic targets that are effective in treating significant cohorts of BAV patients. In addition to the more conventional BAV classifications based on cusp fusion [74,75,76], patient groups identified by shared molecular characteristics, such as excess P-ECM, may elucidate effective therapeutics and biomarkers for BAV.

## Figures and Tables

**Table 1:** Collation of BAV phenotypes in murine models. Gene modifications of mice that exhibit BAVs are listed in order of highest to lowest penetrance of the BAV phenotype, that is, BAV/Total examined. f/f- indicates floxed alleles. The type of BAV fusion events are listed: LC—left coronary cusp; RC—right coronary cusp, NC—non-coronary cusp; nr—not reported. *—56% Males (32/57), 27% Females (7/26); **—20% Males (15/28), 11% Females (12/18). >-P-ECM—excess provisional extracellular matrix is indicated by the following: T—thick valves; AB—increased Alcian blue staining, >H—expanded hinge regions in valve cusps; V—increased immunolocalization of intact Vcan. An X in the alt CNC column indicates altered CNC localization; Alt-TGFβ—indicate models with altered TGFβ signaling; Biomech—refers to genes that are involved in biomechanical signaling when ‘X’ is present; AscAo—abnormalities in the ascending aorta; el—embryonic lethal; Ref—reference of BAV model(s).

BAV Gene(s)	BAV % (BAV/Total)	Fusion	>P-ECM	Alt CNC	Alt TGF	Bio Mech	AscAo	Ref
***Nos3^−/−^;Notch^+/−^***	91% (10/11)	NC-RC	T, AB				X	[38,51]
*Gata5^Cre^*;***Alk2^f/f^***	80% (12/15)	NC-RC	T, >H		X		none	[52]
***Adamts5^−/−^;Smad2^+/−^***	77% (10/13)	NC-LC> NC-RC	T, >H, V		X		X	[53,54]
*Nfatc1^Cre^*;***Ift88^f/f^***	68% (19/28)	NC-RC	AB, T, >H, V			X	nr	[55]
*Isl1^Cre^*;***Gata6^+/^^−^***	56% M, 27% F*	RC-LC	T, AB	X	X		X	[56]
*Nkx2.5^Cre^*;***Jag1^f/f^***	47% (7/15)	NC-RC> RC-LC	>H		X		el	[43]
*Nfatc1^Cre^*;***Exco5^f/f^***	45% (5/11)	NC-RC>RC-LC	>H			X	X	[57]
***Nos3^−/−^***	42% (5/12)	NC-RC		X		X	none	[38,45]
***Rock^DN,^***	42% (13/31)	NC-RC		X			X	[22]
*Isl1^Cre^*;***Vangl2^f/f^***	37% (4/7)	No NC	T	X			nr	[41]
*Tnnt2^Cre^;**Jag1^f/f^;Jag2^f/f^***	36% (4/11)	No NC	>H				nr	[41]
*Nfatc1^en-Cre^;**Notch1^f/f^***	36% (5/14)	NC-RC> RC-LC	T				nr	[43]
*Nfatc1^Cre^*;***Brg1^f/f^***	34% (9/36)	NC-LC	T, AB, V		X		nr	[23]
*Nfatc1^en-Cre^;**Rbpj^f/f^***	33% (2/6)	NC-RC	T				nr	[43]
***Krox20(Egr2)^LacZ/LacZ^***	27% (6/27)	NC-RC> RC-LC	T	X		X	nr	[48]
*Nfatc1^enCre^*;***Jag1^f/f^***	25% (2/8)	NC-RC	>H				nr	[43]
*Tie2^Cre^;**Gata5^f/f^***	25% (7/28)	NC-RC				X	nr	[46]
***Hoxa1^GFP/GFP^***	24% (4/17)	nr		X			el	[58]
*Hoxa3-I^Cre^;**Fgf8^AP/N^***	23% (7/33)	RC-NC		X			X	[59]
***Robo4^tm1Lex/tm1Lex^***	20% M, 11% F**	nr	T				X	[34]
*Nkx2.5^Cre^;**Mib1^f/f^***	17% (1/6)	NC-RC					nr	[43]
***Adamts5^tm1Dgn/tm1Dgn^***	12.5% (3/24)	NC-RC	T, >H, V		X		X	[53,54]
***Matr3^Gt-Ex13/Gt-Ex13^***	11.5% (3/26)	nr		X			X	[60]
***Nkx2-5H^Dneo^***	11%(11/100)	nr			X		nr	[61]
*Wnt1^Cre^;**Krox20^f/f^***	10.5% (2/19)	NC-RC	T				nr	[48]
*Tie2^Cre^;**Krox20^f/f^***	10% (2/20)	nr					nr	[48]
***Npr2^+/−^***	9.4% (6/64)	NC-RC	T, AB				X	[62]
***Notch^+/−^***	8% (1/12)	NC-RC					X	[38,51]
